# Prize Essays

**Published:** 1881-09-20

**Authors:** 


### *PRIZE ESSAYS.*

At the meeting of the Medical Association of Georgia in April last it was agreed to offer three prizes for Essays:

First. A prize of $50 for the best Essay on Medicinal Plants growing in this State, their use in practice, natural history, mode of cultivation and preparation for use and the market.

The second and third Essays, for each of which $25 is to be awarded—may be upon any subject in Medicine or Surgery the writer may choose to adopt.

A committee was appointed to suggest a plan upon which the Essays are to be written. The committee will address a circular to the profession in relation to the subject.

